# Distributional change of women’s adult height in low- and middle-income countries over the past half century: An observational study using cross-sectional survey data

**DOI:** 10.1371/journal.pmed.1002568

**Published:** 2018-05-11

**Authors:** Jewel Gausman, Ivan Meija Guevara, S. V. Subramanian, Fahad Razak

**Affiliations:** 1 Department of Social and Behavioral Sciences, Harvard T. H. Chan School of Public Health, Boston, Massachusetts, United States of America; 2 Women and Health Initiative, Department of Global Health and Population, Harvard T. H. Chan School of Public Health, Boston, Massachusetts, United States of America; 3 Department of Biology, Stanford University, Stanford, California, United States of America; 4 Stanford Center for Population Health Sciences, Stanford University School of Medicine, Stanford, California, United States of America; 5 Department of Demography, University of California at Berkeley, Berkeley, California, United States of America; 6 Harvard Center for Population and Development Studies, Harvard University, Cambridge, Massachusetts, United States of America; 7 St. Michael’s Hospital, Toronto, Ontario, Canada; 8 Institute of Health Policy, Management and Evaluation, University of Toronto, Toronto, Ontario, Canada; 9 Li Ka Shing Knowledge Institute, University of Toronto, Toronto, Ontario, Canada; Umeå Centre for Global Health Research, Umeå University, SWEDEN

## Abstract

**Background:**

Adult height reflects childhood circumstances and is associated with health, longevity, and maternal–fetal outcomes. Mean height is an important population metric, and declines in height have occurred in several low- and middle-income countries, especially in Africa, over the last several decades. This study examines changes at the population level in the distribution of height over time across a broad range of low- and middle-income countries during the past half century.

**Methods and findings:**

The study population comprised 1,122,845 women aged 25–49 years from 59 countries with women’s height measures available from four 10-year birth cohorts from 1950 to 1989 using data from the Demographic and Health Surveys (DHS) collected between 1993 and 2013. Multilevel regression models were used to examine the association between (1) mean height and standard deviation (SD) of height (a population-level measure of inequality) and (2) median height and the 5th and 95th percentiles of height. Mean-difference plots were used to conduct a graphical analysis of shifts in the distribution within countries over time. Overall, 26 countries experienced a significant increase, 26 experienced no significant change, and 7 experienced a significant decline in mean height between the first and last birth cohorts. Rwanda experienced the greatest loss in height (−1.4 cm, 95% CI: −1.84 cm, −0.96 cm) while Colombia experienced the greatest gain in height (2.6 cm, 95% CI: 2.36 cm, 2.84 cm). Between 1950 and 1989, 24 out of 59 countries experienced a significant change in the SD of women’s height, with increased SD in 7 countries—all of which are located in sub-Saharan Africa. The distribution of women’s height has not stayed constant across successive birth cohorts, and regression models suggest there is no evidence of a significant relationship between mean height and the SD of height (β = 0.015 cm, 95% CI: −0.032 cm, 0.061 cm), while there is evidence for a positive association between median height and the 5th percentile (β = 0.915 cm, 95% CI: 0.820 cm, 1.002 cm) and 95th percentile (β = 0.995 cm, 95% CI: 0.925 cm, 1.066 cm) of height. Benin experienced the largest relative expansion in the distribution of height. In Benin, the ratio of variance between the latest and earliest cohort is estimated as 1.5 (95% CI: 1.4, 1.6), while Lesotho and Uganda experienced the greatest relative contraction of the distribution, with the ratio of variance between the latest and earliest cohort estimated as 0.8 (95% CI: 0.7, 0.9) in both countries. Limitations of the study include the representativeness of DHS surveys over time, age-related height loss, and consistency in the measurement of height between surveys.

**Conclusions:**

The findings of this study indicate that the population-level distribution of women’s height does not stay constant in relation to mean changes. Because using mean height as a summary population measure does not capture broader distributional changes, overreliance on the mean may lead investigators to underestimate disparities in the distribution of environmental and nutritional determinants of health.

## Abstract 2

Using dats from Demographic Health Surveys in 59 low- and middle-income countries, Fahad Razak and colleagues examine changes at the population-level in the distribution of height over time.

## Author summary

**Why was this study done?:**

- An adult’s height may provide important insight into childhood health circumstances.
- Height is also related to longevity, mortality from a variety of noncommunicable diseases, maternal mortality, and quality of life.
- Mean height has decreased in some low- and middle-income countries and increased in others, but it is unclear if everyone in the population experiences similar changes in height.
- This paper examines the relationship between changes in the mean of women’s height and the distribution of women’s height across a population.

**What did the researchers do and find?:**

- Data from 59 low- and middle-income countries (*n =* 1,122,845 women, aged 25–49 years) was obtained from the Demographic and Health Surveys, nationally representative surveys conducted at regular intervals around the world.
- Mean-difference plots were used to visually examine changes in mean women’s height in relation to the distribution of height across countries.
- Statistical analyses were conducted to examine the global associations between mean height and the standard deviation of height, as well as median height and the 95th and 5th percentile.
- Seven countries experienced a decline in mean height (all located in sub-Saharan Africa), and 25 countries experienced a change in the standard deviation of height.
- There was an inconsistent relationship between mean height and its standard deviation.

**What do these findings mean?:**

- Changes observed in the distribution of height indicate that transitions in living standards and nutrition have not been experienced equally across many populations. A focus on mean women’s height does not capture these changes.
- Future research on height as a population measure of well-being should include assessment of changes in the distribution as well as the mean.

## Introduction

An individual’s maximum height is both heritable and heavily influenced by childhood environmental exposures [1,2]. Genetics play a limited role in the marginal changes observed in the heights of populations over time, while environmental factors, such as illness and nutritional deprivation, are thought to be the primary determinants [3–8]. Adverse circumstances during periods of rapid growth, such as those occurring in utero [9,10] and during childhood and adolescence [11–15], have been associated with decreased adult height. Height is also associated with future health and well-being. Studies have found that increased height is negatively associated with mortality from a variety of noncommunicable diseases such as cardiac disease, stroke, and some types of cancer [16–21]; adiposity and type 2 diabetes [22]; suicide [23]; and all-cause mortality [24]. Additionally, health-related quality of life [25] and age-related declines in cognitive function [26] are associated with adult height. In India, shorter maternal height is associated with increased child mortality, stunting, and wasting [27]. Short maternal stature has also been associated with increased perinatal mortality [28–30], cesarean delivery [31], and maternal morbidity and mortality [32,33].

Given the sensitivity of height to nutritional and disease exposures, examining changes in adult height over time may provide insight into the underlying health status of a population. Two recent studies have found that mean height is declining in several countries in sub-Saharan Africa (SSA), while it has generally increased across the European, Eastern Mediterranean, South-East Asian, and the Western Pacific regions [34–36]. Another study found that mean height has stagnated among men in some countries in Central Asia, the Middle East, and North Africa, while women have continued to grow taller [36]. In addition, mean height has served as a crucial measure of overall human health in a large body of economic work, for example Robert Fogel’s research on economic history and Angus Deaton’s research on consumption, poverty, and welfare [3,7,8].

Global trends in height [3,34–41], and many population-level health metrics [42–45], are frequently examined by analyzing changes in the mean without considering other properties, such as variance. Additionally, progress towards achievement of the Millennium Development Goals in nutrition is often discussed in terms of changes in mean height within populations [46,47]. The use of mean levels makes an important assumption that as the average level in the population changes, the dispersion around the mean remains constant, making the mean a good proxy for population-level changes [2]. Recent research has found that this assumption does not apply to body weight, where rising mean BMI is accompanied by a widening of the distribution, with gains concentrated among the highest percentiles of the BMI distribution and relatively little change among individuals who are underweight [48,49]. This has direct equity implications for use of the mean to measure population health, since underweight individuals would be expected to benefit from weight gain, whereas overweight individuals would benefit from weight loss.

In this paper, we examine whether relying on the mean as a summary measure of height may obscure significant changes in the distribution of height over time both within and across populations. We describe changes in mean height across birth cohorts, and explore whether there is an association between change in mean height and its distribution, by systematically examining the mean and standard deviation (SD) of women’s height both within and across a broad range of low- and middle-income countries (LMICs) over the past half century. We also examine the change observed in the left and right tails of the height distribution to understand distributional change over time.

## Methods

We include the prospective analysis plan used to guide our analysis as S1 Analysis Plan. This study is reported as per the Strengthening the Reporting of Observational Studies in Epidemiology (STROBE) guidelines (S1 STROBE Checklist) [50]. The study is based on an anonymous, publicly available dataset with no personally identifiable information. Ethical approval for the Demographic and Health Surveys (DHS) was provided centrally by the ORC Macro Institutional Review Board and by individual review boards within each participating country.

### Data sources

We used data from DHS surveys, which are nationally representative, cross-sectional household surveys that have been conducted regularly in more than 85 countries since 1984 [51,52]. DHS surveys are comparable over time and across countries, as DHS employs standardized interviewer training, measurement tools, data collection techniques, and questionnaires [53]. A multistage stratified cluster design that samples households within a selection of primary sampling units ensures that sampled households are nationally representative [54]. The final sample size typically ranges from 5,000 to 30,000 households [52].

The primary objective of DHS is to collect data on basic demographic and health indicators [54]. Anthropometric measurement for a specified subsample of women aged 15–49 years was initially included in DHS surveys in the early 1990s; thus, some surveys include women’s height for women born as early as 1940 [55]. In order to ensure accuracy in height measurements, height is measured by 2 well-trained individuals who have completed a standardized training process, and is measured to the nearest millimeter using a measuring board with a headpiece [56]. The method used for measuring height in DHS surveys was first introduced in 1987 and has remained consistent since its introduction [57]. Additionally, anthropometric data reported by DHS undergo regular quality assessment to ensure both their validity and reliability over time [58].

### Study population and sample

The study population comprises 1,122,845 women aged 25–49 years from 59 countries with women’s height measures available from survey waves spanning from 1993 to 2013. Women aged less than 25 years were excluded from the sample in order to ensure that full height attainment had been achieved in order to limit potential bias [35]. Women with missing height values (*n =* 18,802), those excluded from the one-third or 50% subsample of women selected for anthropometric assessment (*n =* 246,276), and those who had implausible values recorded for height (less than 100 cm or greater than 200 cm) (*n =* 714) were also excluded from the study [35].

### Outcome and exposure

Height (in centimeters) was specified as a continuous outcome. Using methods described previously, the SD and the 5th and 95th percentiles were used as outcomes in statistical modeling because they provide information on how the distribution of height changes between and within countries across birth cohorts [48,49].

The primary exposure of interest was birth cohort. Women in each country were divided into 4 birth cohorts: 1950–1959, 1960–1969, 1970–1979, and 1980–1989. Several steps were taken to limit bias within birth cohorts. While we had initially planned to include women born before 1950 from all countries in our analysis, we made 2 data-driven changes from our original analysis plan after assembling the data. As so few women in the sample were born before 1950 (*n =* 3,194 across all 59 countries), we excluded them from the analysis because the sample sizes of birth cohorts born before 1950 were too small within each country to offer meaningful comparisons. Similarly, birth cohorts that contained fewer than 500 women were also excluded from the analysis because they were found to be too small for robust analyses of distributional changes and introduced instability into the models, especially around the tails (5th and 95th percentile) of the distribution (*n =* 5,641).

### Analysis

For global models, we pooled data across birth cohorts and countries. A second dataset was also created that contained the percentile values for the height distribution in each country for each birth cohort. Stata version 14 [59] was used to conduct the statistical analysis. Graphics were produced using the statistical package R [60].

### Modeling of distributional parameters

Multilevel regression was used to account for the nested structure of the data, as the outcome measures of interest (SD, 5th percentile, and 95th percentile) are nested within cohort *i* at level 1 which is nested in country *j* at level 2.

For example, the general statistical model at level 1 for the SD of height is specified as

$$Y_{ij}=\beta_{0j}+\beta_{1}{height}_{ij}+e_{ij}$$

where *Y*_*ij*_ is the SD of height in cohort *i* in country *j*, β_0*j*_ is the overall SD of height in each country, β_1_ is the slope of the relationship between mean height and SD of height, height_*ij*_ is the mean height in cohort *i* in country *j*, and *e*_*ij*_ is the residual for cohort *i* in country *j*.

The model at level 2 is represented as

$$\beta_{oj}=\beta_{0}+u_{0j}$$

where β_0_ is the grand mean SD of height across all countries and *u*_0*j*_ is the residual of the mean SD of height for country *j* from the overall SD of height. Both models are combined to obtain

$$Y_{ij}=\beta_{0}+\beta_{1}{height}_{ij}+(u_{0j}+e_{ij})$$

Using data from all countries and cohorts, multilevel regression models were constructed to examine the association between (1) mean height and SD of height and (2) median height and the 5th and 95th percentiles of height.

Additional tests were conducted to determine whether country-level changes in the SD were significant across birth cohorts. The ratio of the variance in height between the latest cohort versus the earliest cohort was calculated, and 95% CIs were estimated. For each country, an F-test for equality of variance was performed to test the change in variance (SD^2^) between the earliest and most recent birth cohort [61]. Additionally, a *t* test was performed to formally evaluate whether the change in mean was significant between these same cohorts in each country.

### Graphical analyses

As quantitative approaches to examining changes in distributions remain limited, we also employed a graphical approach. Mean-difference (MD) plots enable a visual comparison of 2 distributions by plotting the differences between corresponding quantiles from the 2 distributions on the *y*-axis against the means of the same quantiles on the *x*-axis [62,63]. On these plots, a point’s location in relation to 0 on the *y*-axis illustrates the direction and magnitude of the change in value of that point in one distribution compared to its value in the other [63]. In other words, if the 2 distributions being compared are equal, the resulting plot is a horizontal line at the value 0. An upward sloping line indicates increasing dispersion in the distribution while a downward sloping line suggests contraction.

In our analysis, we created a MD plot for each country using data from the earliest and latest available birth cohorts to visually illustrate the shifts in the distribution of height. Points that fall below the horizontal line drawn at 0 indicate losses in height at that place in the distribution, while points that fall above the line indicate gains in height at that place in the distribution.

## Results

The final analytic sample comprised 857,053 women. Table 1 provides basic statistics on the population included in the study according to country and birth cohort, as well as change in mean height and SD of height for each country. While 26 countries exhibited statistically significant gains in mean height across birth cohorts, 7 countries experienced statistically significant declines, all of which are in SSA. Rwanda experienced the largest decline in mean height (−1.4 cm, 95% CI: −1.8, 1.0), from 158.0 cm among the cohort born in 1950–1959 to 156.6 cm among the cohort born in 1980–1989, while Colombia experienced the greatest gain in height (2.6 cm, 95% CI: 2.4, 2.8) from 143.8 cm among the cohort born in the years 1950–1959 to 143.6 cm among the cohort born in the years 1980–1989. Sierra Leone consistently had the largest spread in the height distribution, with a SD that ranged from 11.6 to 11.8 cm depending on the birth cohort. Benin experienced the largest relative expansion in the distribution of height, from a SD of 6.4 cm among the cohort born in the years 1950–1959 to 7.9 cm among the cohort born in the years 1980–1989; thus, the ratio of the SD among the latest versus the earliest birth cohorts was 1.52 (95% CI: 1.4, 1.6), while Uganda and Lesotho experienced the largest relative contraction in SD, with a ratio comparing the latest versus the earliest cohorts estimated to be 0.8 (95% CI: 0.7, 0.9) in both countries.

**Table 1:** Women’s height across countries by birth cohort (women aged 25–49 years).

Country	Survey years	Birth cohort	*n* for birth cohort	Height (in centimeters)					Change in mean (latest minus earliest cohort)^§^ (95% CI)	Ratio of variance (SD^2^) in height (latest/earliest cohort) (95% CI)^§§^
				Mean	SD	5th percentile	50th percentile	95th percentile		
Albania	2008	1960–1969	2,338	160.4	6.7	149.9	160.2	170.5	0.70** (0.11, 1.29)	1.03 (0.91, 1.17)
		1970–1979	1,916	160.9	6.8	150.1	160.7	172.0		
		1980–1989	658	161.1	6.8	150.4	161.8	170.5		
Armenia	2000, 2005	1950–1959	2,231	157.5	6.2	147.6	157.5	168.0	1.00*** (0.65, 1.35)	0.88*** (0.81, 0.95)
		1960–1969	3,516	157.8	5.7	148.3	158.0	167.1		
		1970–1979	2,383	158.5	5.8	148.6	158.6	167.8		
Azerbaijan	2006	1950–1959	522	158.1	6.0	147.7	158.5	168.2	1.00*** (0.43, 1.57)	0.87* (0.76, 1.00)
		1960–1969	2,368	158.5	6.0	149.1	159.1	168.2		
		1970–1979	2,052	159.1	5.6	149.8	159.1	168.4		
Bangladesh	1996, 1999, 2004, 2007, 2011	1950–1959	2,040	149.9	5.5	141.0	149.7	158.9	1.00*** (0.72, 1.28)	1.00 (0.93, 1.07)
		1960–1969	10,426	150.4	5.5	141.7	150.3	159.2		
		1970–1979	13,832	150.7	5.6	141.8	150.6	159.6		
		1980–1989	5,674	150.9	5.5	142.0	150.8	159.7		
Benin	1996, 2001, 2006, 2011	1950–1959	1,669	158.7	6.4	148.7	158.8	169.0	0.70*** (0.34, 1.06)	1.52*** (1.41, 1.64)
		1960–1969	7,337	159.8	7.0	149.5	160.0	170.5		
		1970–1979	11,798	159.9	7.1	149.5	160.0	170.5		
		1980–1989	6,315	159.4	7.9	148.6	159.6	170.0		
Bolivia	1993, 1998, 2003, 2008	1950–1959	3,255	150.6	5.9	141.5	150.2	160.3	2.20*** (1.87, 2.53)	1.07* (0.99, 1.15)
		1960–1969	10,396	151.3	5.9	142.2	151.0	161.1		
		1970–1979	9,937	151.8	5.9	142.8	151.5	161.7		
		1980–1989	2,174	152.8	6.1	143.4	152.5	163.0		
Burkina Faso	1998, 2003, 2010	1950–1959	1,747	161.4	6.1	151.4	161.5	171.0	0.40** (0.01, 0.79)	0.90* (0.82, 0.99)
		1960–1969	5,382	161.8	6.2	152.2	161.7	171.6		
		1970–1979	6,197	161.8	6.0	152.0	162.0	171.5		
		1980–1989	1,858	161.8	5.8	152.3	161.9	171.5		
Burundi	2010	1960–1969	587	155.8	6.7	146.0	155.7	166.4	−0.50 (−1.17, 0.17)	0.88* (0.76, 1.02)
		1970–1979	949	155.9	6.9	145.7	155.9	166.3		
		1980–1989	1,000	155.3	6.3	145.1	155.0	166.3		
Cambodia	2000, 2005, 2010	1950–1959	2,048	152.7	5.2	144.5	152.8	161.0	0.10* (−0.21, 0.41)	1.00 (0.92, 1.09)
		1960–1969	6,075	152.9	5.4	144.3	152.9	161.5		
		1970–1979	5,302	152.8	5.3	144.2	152.8	161.5		
		1980–1989	2,316	152.8	5.2	144.5	152.8	161.5		
Cameroon	1998, 2004, 2011	1960–1969	2,455	160.6	6.2	150.5	160.6	170.3	−0.10 (−0.49, 0.29)	1.17*** (1.07, 1.27)
		1970–1979	3,577	160.7	6.7	150.5	160.7	171.1		
		1980–1989	1,861	160.5	6.7	150.3	160.5	171.4		
Chad	1996, 2004	1950–1959	641	163.2	6.2	153.1	163.2	173.0	−0.70*** (−1.25, −0.15)	1.00 (0.88, 1.13)
		1960–1969	2,410	163.0	6.4	152.7	162.9	173.6		
		1970–1979	2,162	162.5	6.2	152.0	162.4	172.3		
Colombia	1995, 2000, 2004, 2006	1950–1959	4,523	153.7	6.3	143.8	153.7	163.9	2.60*** (2.36, 2.84)	0.97 (0.92, 1.02)
		1960–1969	22,988	154.8	6.1	145.0	154.7	164.9		
		1970–1979	23,502	155.5	6.1	145.8	155.4	165.4		
		1980–1989	6,770	156.3	6.2	146.3	156.4	166.4		
Congo (Brazzaville)	2005, 2011	1960–1969	1,980	158.8	7.5	147.0	159.0	170.2	0.50* (0.05, 0.96)	0.73*** (0.67, 0.78)
		1970–1979	3,568	158.8	7.4	146.8	159.0	170.2		
		1980–1989	1,602	159.3	6.4	149.0	159.2	170.0		
Congo, Democratic Republic	2007, 2013	1960–1969	1,643	157.7	7.7	145.2	157.8	169.5	−0.70*** (−1.15, −0.25)	0.90** (0.83, 0.98)
		1970–1979	3,191	157.8	7.8	146.4	157.6	170.0		
		1980–1989	3,340	157	7.3	145.2	157.0	168.4		
Côte d’Ivoire	1994, 1998, 2011	1950–1959	935	158.8	5.9	149.0	159.0	168.0	0.20 (−0.33, 0.73)	1.18** (1.04, 1.33)
		1960–1969	2,898	159.2	6.0	149.5	159.0	169.1		
		1970–1979	1,522	159.4	6.7	149.3	159.1	170.0		
		1980–1989	1,170	159.0	6.4	148.9	159.0	169.3		
Dominican Republic	1996	1950–1959	1,632	156.0	6.2	146.0	155.9	166.4	0.30 (−0.33, 0.93)	1.07 (0.93, 1.23)
		1960–1969	2,281	156.9	6.2	146.8	156.8	167.5		
		1970–1979	519	156.3	6.4	146.5	155.9	167.2		
East Timor	2009	1960–1969	2,453	150.9	5.7	141.5	150.6	160.0	−0.30 (−0.64, 0.04)	0.93 (0.86, 1.01)
		1970–1979	3,163	150.8	5.7	142.0	150.6	159.5		
		1980–1989	1,846	150.6	5.5	142.0	150.4	159.5		
Egypt	1995, 2000, 2008,	1950–1959	7,281	157.8	5.9	148.1	158.1	167.4	2.00*** (1.77, 2.23)	0.93** (0.88, 0.99)
		1960–1969	19,744	158.3	5.9	148.6	158.4	168.0		
		1970–1979	16,297	158.9	5.8	149.2	159.0	168.3		
		1980–1989	3,660	159.8	5.7	150.6	159.6	169.2		
Ethiopia	2005, 2010	1950–1959	2,376	156.3	5.9	146.5	156.4	166.1	0.30* (0.00, 0.60)	1.10** (1.03, 1.18)
		1960–1969	6,327	156.5	6.1	146.8	156.3	166.8		
		1970–1979	8,982	156.4	6.5	146.2	156.3	166.6		
		1980–1989	4,411	156.6	6.2	146.4	156.5	166.3		
Gabon	2000, 2012	1960–1969	1,469	159.3	6.4	149.3	159.2	169.9	1.20* (0.73, 1.67)	0.94 (0.84, 1.04)
		1970–1979	2,017	159.6	6.2	149.8	159.4	169.8		
		1980–1989	1,274	160.5	6.2	150.1	160.9	171.0		
Ghana	1993, 1998, 2003, 2008	1950–1959	1,383	158.9	6.3	149.2	158.6	169.1	0.60 (0.02, 1.18)	1.03 (0.91, 1.18)
		1960–1969	3,949	159.1	6.3	149.4	158.9	169.2		
		1970–1979	3,388	159.1	6.6	149.5	158.9	169.4		
		1980–1989	683	159.5	6.4	150.1	159.2	169.8		
Guatemala	1995	1950–1959	1,109	147.0	6.7	137.6	146.5	158.0	1.30*** (0.82, 1.78)	0.97 (0.88, 1.07)
		1960–1969	2,331	148.3	6.6	137.7	148.0	159.8		
Guinea	1999, 2005, 2012	1950–1959	634	158.8	6.4	149.7	158.7	168.8	0.8** (0.20, 1.40)	1.06 (0.93, 1.21)
		1960–1969	2,690	159.0	6.6	148.9	159.2	168.6		
		1970–1979	3,245	159.1	6.3	149.2	159.0	169.2		
		1980–1989	1,458	159.6	6.6	149.4	159.7	169.9		
Guyana	2009	1960–1969	1,132	157.2	7.2	145.9	157.0	169.2	0.00 (−0.70, 0.70)	1.00 (0.87, 1.15)
		1970–1979	1,299	157.4	7.3	146.1	157.2	169.5		
		1980–1989	631	157.2	7.2	145.8	157.3	168.6		
Haiti	1994, 2000, 2005, 2012	1950–1959	2,541	157.8	6.6	147.0	158.0	168.0	2.00*** (1.64, 2.36)	0.91** (0.84, 0.99)
		1960–1969	5,442	158.6	6.4	148.5	158.6	168.7		
		1970–1979	5,089	159.0	6.5	148.7	159.0	169.5		
		1980–1989	2,450	159.8	6.3	149.6	159.8	170.3		
Honduras	2005, 2011	1950–1959	1,167	152.2	6.0	142.4	152.3	162.6	1.30*** (0.92, 1.68)	1.10** (1.01, 1.20)
		1960–1969	6,947	152.4	6.1	142.4	152.4	162.5		
		1970–1979	10,815	152.8	6.2	142.8	152.8	162.9		
		1980–1989	5,455	153.5	6.3	143.4	153.5	163.7		
India	1998, 2005	1950–1959	25,609	151.1	5.9	141.7	151.0	160.5	0.90*** (0.72, 1.08)	1.00 (0.96, 1.04)
		1960–1969	55,092	151.6	5.9	142.3	151.6	161.2		
		1970–1979	50,422	151.8	5.9	142.4	151.8	161.5		
		1980–1989	4,846	152.0	5.9	142.3	151.8	161.5		
Jordan	1997, 2002, 2007, 2012	1950–1959	1,665	156.6	6.1	147.0	156.5	166.5	2.50*** (2.14, 2.86)	0.90** (0.83, 0.98)
		1960–1969	6,753	157.7	6.1	148.5	157.6	167.1		
		1970–1979	7,376	158.4	5.9	149.0	158.4	168.0		
		1980–1989	2,740	159.1	5.8	150.0	158.9	168.3		
Kazakhstan	1995, 1999	1950–1959	1,586	158.4	6.1	148.4	158.6	168.2	1.10*** (0.69, 1.51)	1.00 (0.91, 1.10)
		1960–1969	1,779	159.5	6.1	149.5	159.3	170.0		
Kenya	1993, 1998, 2003, 2008	1950–1959	1,802	158.9	6.5	149.1	158.5	169.8	0.60 (0.12, 1.08)	1.03 (0.93, 1.14)
		1960–1969	5,900	159.5	6.8	149.6	159.3	169.9		
		1970–1979	5,124	160.0	6.7	149.8	160.0	170.7		
		1980–1989	1,175	159.5	6.6	150.3	159.4	169.9		
Kyrgyz Republic	1997, 2012	1950–1959	827	157.4	5.7	148.5	157.2	167.5	2.40*** (1.93, 2.87)	1.04 (0.92, 1.16)
		1960–1969	2,388	158.7	6.1	149.2	158.7	169.0		
		1970–1979	2,234	159.6	5.8	150.4	159.4	169.2		
		1980–1989	1,914	159.8	5.8	150.5	159.8	169.7		
Lesotho	2004, 2009	1960–1969	1,381	157.5	7.0	148.0	157.2	168.1	−0.70* (−1.32, −0.08)	0.81*** (0.71, 0.93)
		1970–1979	1,805	157.8	6.5	147.3	157.8	168.2		
		1980–1989	602	156.8	6.3	148.2	156.6	166.7		
Liberia	2006, 2013	1960–1969	1,875	158.0	6.3	148.2	157.7	168.5	−0.4*** (−0.81, 0.01)	1.06 (0.97, 1.17)
		1970–1979	3,117	157.8	6.3	148.2	157.6	168.1		
		1980–1989	1,824	157.6	6.5	147.4	157.4	167.7		
Madagascar	1997, 2003, 2008	1950–1959	1,225	154.1	6.0	144.6	154.0	163.3	−1.00*** (−1.48, −0.52)	0.97 (0.86, 1.08)
		1960–1969	4,428	154.4	5.9	145.2	154.3	164.2		
		1970–1979	4,969	153.6	6.0	144.1	153.5	163.7		
		1980–1989	1,109	153.1	5.9	143.2	152.8	163.0		
Malawi	2000, 2004, 2010	1950–1959	2,346	156.1	6.0	146.7	156.0	166.0	0.20 (−0.18, 0.58)	1.07 (0.98, 1.17)
		1960–1969	5,779	156.4	6.2	147.0	156.1	166.5		
		1970–1979	8,058	156.3	6.4	146.5	156.1	166.6		
		1980–1989	1,700	156.3	6.2	147.1	156.3	166.3		
Maldives	2009	1960–1969	1,376	149.7	5.8	140.5	149.5	158.9	2.50*** (2.06, 2.94)	0.93 (0.84, 1.04)
		1970–1979	1,917	151.5	5.6	142.5	151.5	160.9		
		1980–1989	1,203	152.2	5.6	143.5	152.2	161.2		
Mali	1995, 2001, 2006	1950–1959	2,730	161.7	6.1	152.0	161.6	171.5	−0.70*** (−1.13, −0.27)	1.21*** (1.10, 1.33)
		1960–1969	8,060	161.6	6.2	152.0	161.5	171.6		
		1970–1979	7,429	161.5	6.5	151.3	161.5	171.7		
		1980–1989	1,309	161.0	6.7	150.8	161.2	171.1		
Moldova	2005	1950–1959	891	160.0	6.1	150.0	160.0	170.0	1.50*** (1.01, 1.99)	1.03 (0.92, 1.16)
		1960–1969	1,864	160.7	6.2	150.5	161.0	170.5		
		1970–1979	1,799	161.5	6.2	152.0	161.4	172.0		
Morocco	2003	1950–1959	1,983	157.6	5.9	148.0	157.5	168.0	1.20*** (0.89, 1.51)	1.03 (0.96, 1.11)
		1960–1969	3,950	158.6	6.0	149.0	158.2	168.5		
		1970–1979	4,401	158.8	6.0	150.0	158.5	169.0		
Mozambique	1997, 2003, 2011	1950–1959	1,417	155.7	6.3	146.2	155.7	166.8	−0.20 (−0.59, 0.19)	0.91** (0.83, 0.99)
		1960–1969	5,348	156.0	6.0	146.5	156.0	166.0		
		1970–1979	7,168	155.4	6.3	145.2	155.5	165.7		
		1980–1989	3,187	155.5	6.0	146.0	155.5	165.5		
Namibia	2006, 2013	1960–1969	2,285	161.1	7.0	149.8	161.1	172.0	0.20 (−0.23, 0.63)	0.94 (0.86, 1.03)
		1970–1979	3,775	161.2	6.8	150.4	161.3	171.5		
		1980–1989	1,781	161.3	6.8	150.2	161.4	172.1		
Nepal	1996, 2001, 2006, 2011	1950–1959	2,250	149.5	5.5	140.5	149.6	158.4	1.60*** (1.27, 1.93)	1.04 (0.95, 1.13)
		1960–1969	6,847	150.4	5.5	141.7	150.4	159.3		
		1970–1979	7,037	150.9	5.6	142.1	150.9	160.0		
		1980–1989	2,122	151.1	5.6	141.8	151.3	159.8		
Nicaragua	1997, 2001	1950–1959	4,012	153.6	6.0	144.3	153.5	163.4	0.30 (0.03, 0.57)	1.00 (0.94, 1.06)
		1960–1969	6,607	154.0	6.1	144.6	154.0	163.5		
		1970–1979	3,859	153.9	6.0	144.6	153.8	163.4		
Niger	1998, 2006, 2012	1960–1969	2,724	160.6	6.0	151.0	160.4	170.5	−0.20 (−0.56, 0.16)	1.17*** (1.08, 1.27)
		1970–1979	3,359	160.6	5.9	151.3	160.5	170.3		
		1980–1989	2,128	160.4	6.5	150.2	160.2	171.0		
Nigeria	1999, 2003, 2008	1950–1959	1,303	157.9	7.7	147.0	157.8	170.0	0.00 (−0.46, 0.46)	0.92* (0.85, 1.00)
		1960–1969	8,030	159.0	7.7	148.0	158.9	170.6		
		1970–1979	11,396	158.8	7.3	148.0	158.9	170.0		
		1980–1989	5,512	157.9	7.4	147.1	158.1	169.0		
Pakistan	2012	1960–1969	798	154.4	6.1	145.2	154.5	163.8	0.20 (−0.32, 0.72)	0.94 (0.83, 1.06)
		1970–1979	1,441	154.7	6.2	145.0	155.0	165.1		
		1980–1989	1,505	154.6	5.9	145.4	154.5	164.0		
Peru	1996, 2000	1950–1959	6,544	150.0	6.0	140.9	149.8	160.0	1.40*** (1.19, 1.61)	0.97 (0.92, 1.02)
		1960–1969	12,131	150.7	5.7	141.9	150.5	160.3		
		1970–1979	6,157	151.4	5.9	142.4	151.0	161.5		
Rwanda	2000, 2005, 2010	1950–1959	1,795	158.0	6.8	147.5	157.6	169.1	−1.40*** (−1.84, −0.96)	0.86*** (0.78, 0.94)
		1960–1969	4,373	158.1	6.5	148.0	158.0	169.0		
		1970–1979	5,008	157.5	6.5	147.6	157.4	168.1		
		1980–1989	1,691	156.6	6.3	146.7	156.5	167.0		
Sao Tome and Principe	2008	1960–1969	515	158.7	9.2	147.0	160.0	170.0	0.80 (−0.39, 1.99)	0.85 (0.71, 1.04)
		1970–1979	693	159.8	8.1	150.0	160.0	172.0		
		1980–1989	355	159.5	8.5	150.0	160.0	170.0		
Senegal	2005, 2010	1960–1969	1,535	163.2	6.7	152.5	163.0	174.0	0.70 (0.22, 1.18)	1.00 (0.90, 1.11)
		1970–1979	2,573	163.4	6.8	153.0	163.4	173.7		
		1980–1989	1,447	163.9	6.7	153.5	164.0	175.2		
Sierra Leone	2008	1960–1969	604	156.0	11.8	137.1	157.0	173.6	−0.70 (−1.99, 0.59)	0.98 (0.84, 1.15)
		1970–1979	1,064	155.6	11.6	136.3	156.3	174.0		
		1980–1989	679	155.3	11.7	135.2	156.1	174.5		
Swaziland	2006	1960–1969	890	158.9	6.2	148.3	158.8	169.0	0.50 (−0.03, 1.03)	0.94 (0.83, 1.06)
		1970–1979	1,204	159.4	6.0	149.8	159.4	169.2		
Tajikistan	2012	1960–1969	1,315	158.3	5.9	150.0	158.0	168.4	0.10 (−0.30, 0.50)	1.00 (0.91, 1.10)
		1970–1979	2,112	158.6	6.0	149.2	158.4	168.5		
		1980–1989	2,312	158.4	5.9	149.5	158.3	167.8		
Tanzania	1996, 2004, 2009	1950–1959	1,368	156.0	6.4	145.7	156.0	166.5	0.20 (−0.25, 0.65)	0.91* (0.82, 1.01)
		1960–1969	5,462	156.6	6.3	146.5	156.4	166.8		
		1970–1979	6,397	156.8	6.3	146.6	156.5	167.2		
		1980–1989	1,598	156.2	6.1	146.5	156.1	166.3		
Turkey	1998, 2003	1960–1969	1,574	155.9	5.6	147.0	155.8	165.5	0.80*** (0.44, 1.16)	1.07 (0.98, 1.17)
		1970–1979	2,395	156.7	5.8	147.5	156.6	166.2		
Uganda	1995, 1996, 2011	1950–1959	1,266	158.6	7.0	147.2	158.9	170.0	0.20 (−0.37, 0.77)	0.81*** (0.72, 0.92)
		1960–1969	3,777	159.1	6.4	149.2	159.0	170.0		
		1970–1979	3,227	158.7	6.6	148.4	158.6	169.3		
		1980–1989	884	158.8	6.3	149.0	159.0	169.1		
Uzbekistan	1996	1950–1959	916	159.3	6.2	149.4	159.1	169.9	0.10 (−0.44, 0.64)	1.10 (0.97, 1.24)
		1960–1969	1,224	159.4	6.5	150.0	159.1	170.0		
Zambia	1996, 2001, 2007	1950–1959	1,604	158.3	6.3	148.2	158.4	168.5	−0.50 (−1.05, 0.05)	1.17*** (1.04, 1.31)
		1960–1969	4,267	158.8	6.2	149.0	158.6	169.0		
		1970–1979	4,166	158.3	6.4	148.2	158.2	168.5		
		1980–1989	856	157.8	6.8	146.3	158.0	168.0		
Zimbabwe	1994, 1999, 2005, 2010	1950–1959	1,657	160.1	6.6	150.1	160.0	171.0	0.10 (−0.27, 0.47)	0.85*** (0.79, 0.93)
		1960–1969	5,856	160.0	6.2	150.0	160.0	170.1		
		1970–1979	7,726	160.4	6.3	150.9	160.4	170.5		
		1980–1989	4,142	160.2	6.1	150.5	160.2	169.8		

^§^A *t* test was used to evaluate changes in mean across birth cohorts.

^§§^A F-test of equality of variance was used to evaluate changes in variance across birth cohorts.

*p-value ≤ 0.05

**p-value ≤ 0.01

***p-value ≤ 0.001

**Fig 1:**
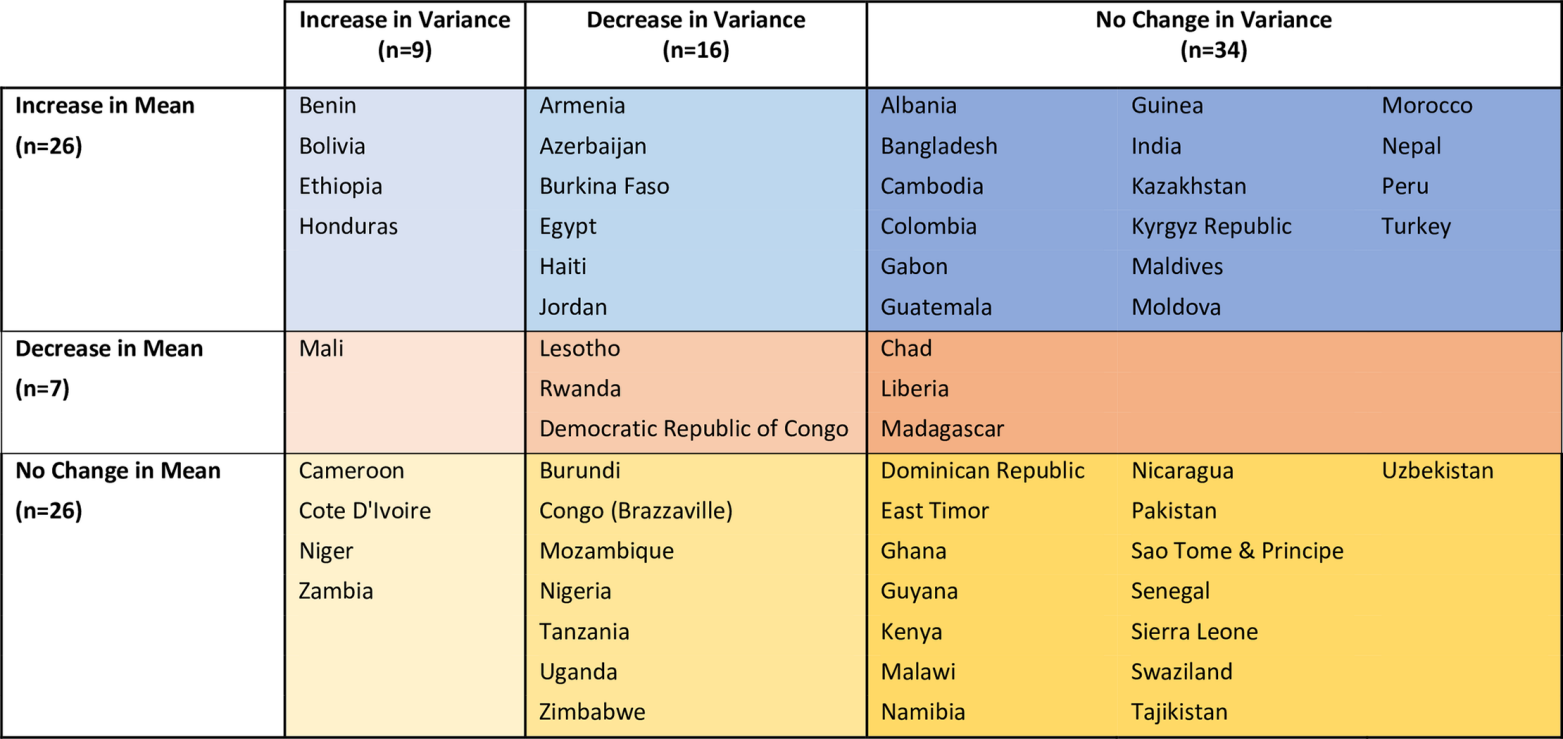
Summary of changes in mean and variance of women’s height from first to last birth cohort by country.

Across countries, changes in the SD of height in relation to changes in mean height are complex and varied. Fig 1 summarizes the results presented in Table 1 regarding the observed change in mean and SD. Overall, 34 of the 59 countries included in the study experienced no significant change in the variance of height between the first and the last birth cohort. Sixteen of these countries experienced increases in mean height, 3 countries experienced decreases in mean height, and 15 countries experienced no significant change in either mean height or the SD of height.

Nearly approximately 40% of the countries included in the study (25 out of 59) experienced a significant change in the SD of height between the first and last birth cohorts (9 experienced increased dispersion and 16 experienced decreased dispersion). Of the 9 countries where variance increased, 4 exhibited an increase, 1 exhibited a decrease, and 4 exhibited no change in mean height, and all but 2 are in SSA. There was more geographic diversity in the countries in which the SD of height decreased, including countries in South America, Africa, Eastern Europe, and South Asia. Six of these countries experienced increases in mean height, 3 experienced decreases in mean height, and 7 experienced no significant change in mean height.

**Fig 2:**
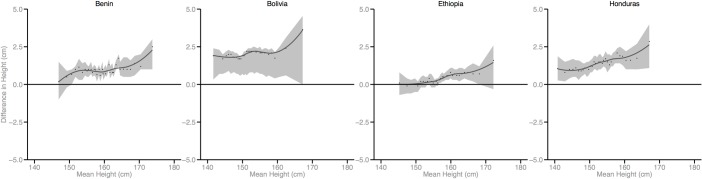
Mean-difference plots by country: Countries with increased variance and increased mean height (95% confidence interval shaded).

**Fig 3:**
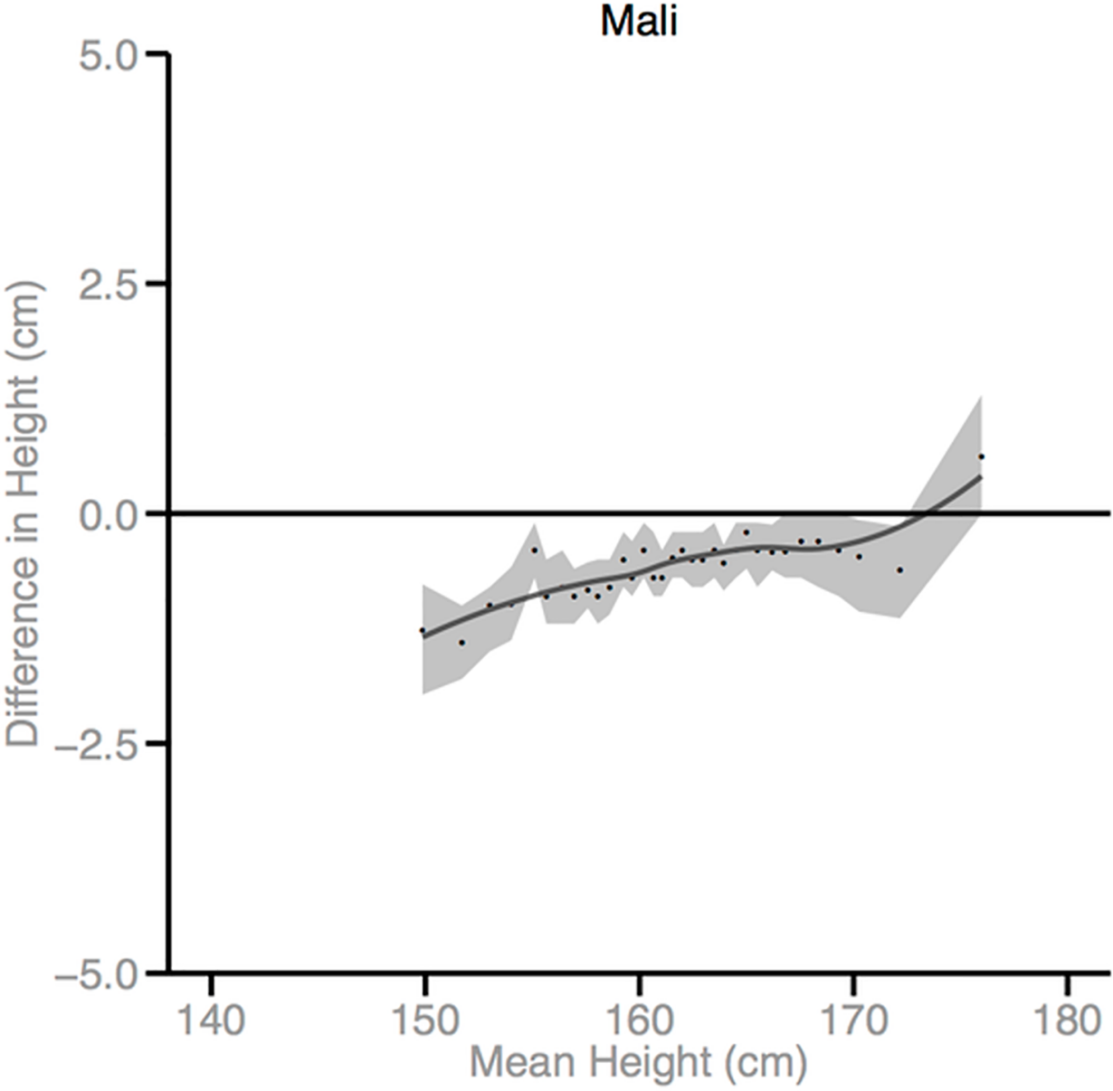
Mean-difference plots by country: Countries with increased variance and decreased mean height (95% confidence interval shaded).

**Fig 4:**
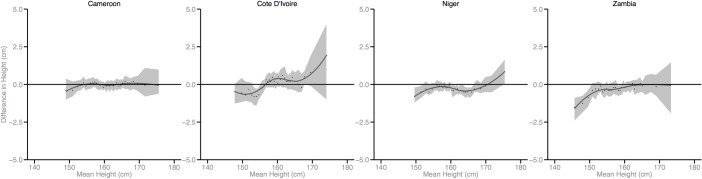
Mean-difference plots by country: Countries with increased variance and no change in mean height (95% confidence interval shaded).

**Fig 5:**
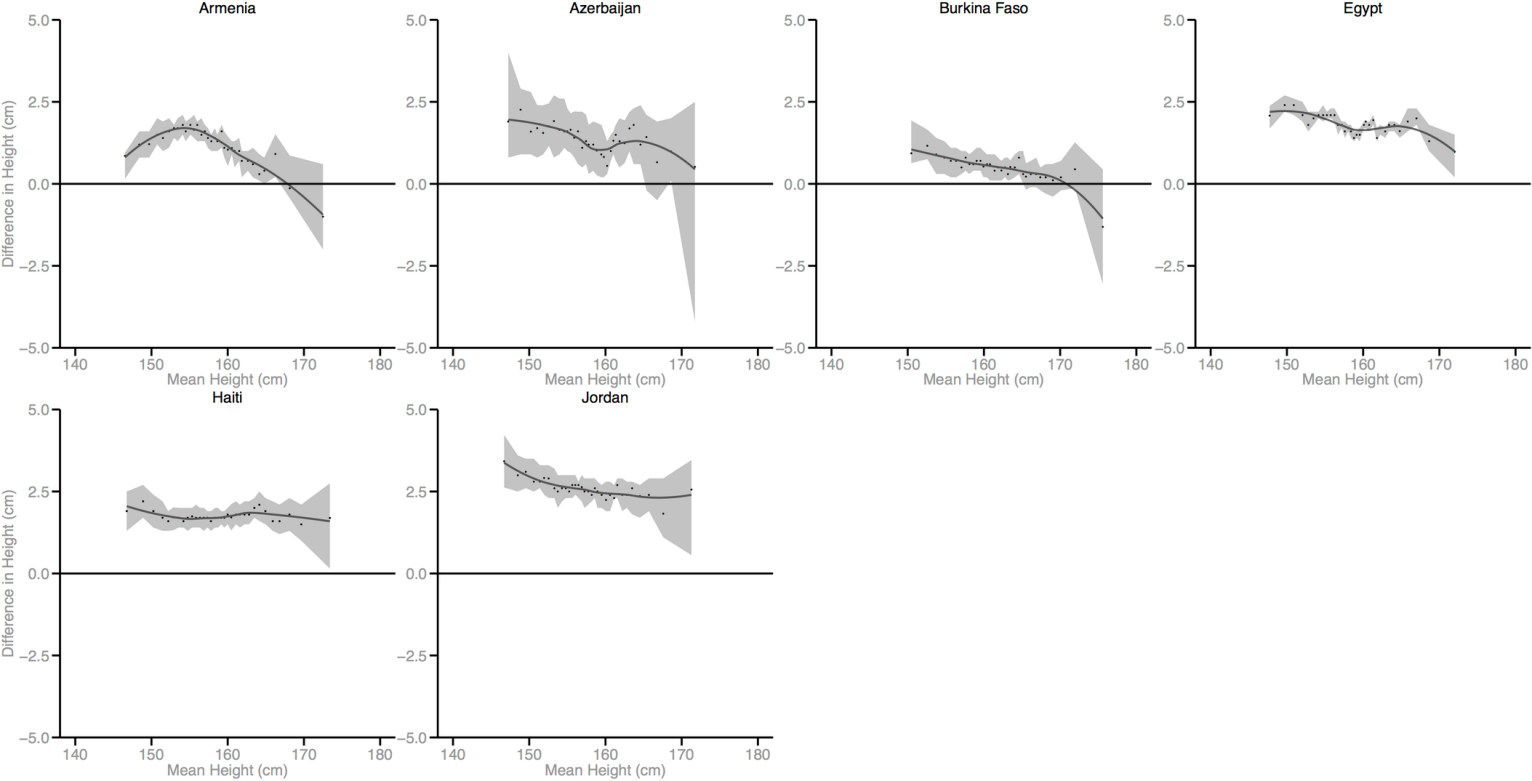
Mean-difference plots by country: Countries with decreased variance and increased mean height (95% confidence interval shaded).

**Fig 6:**
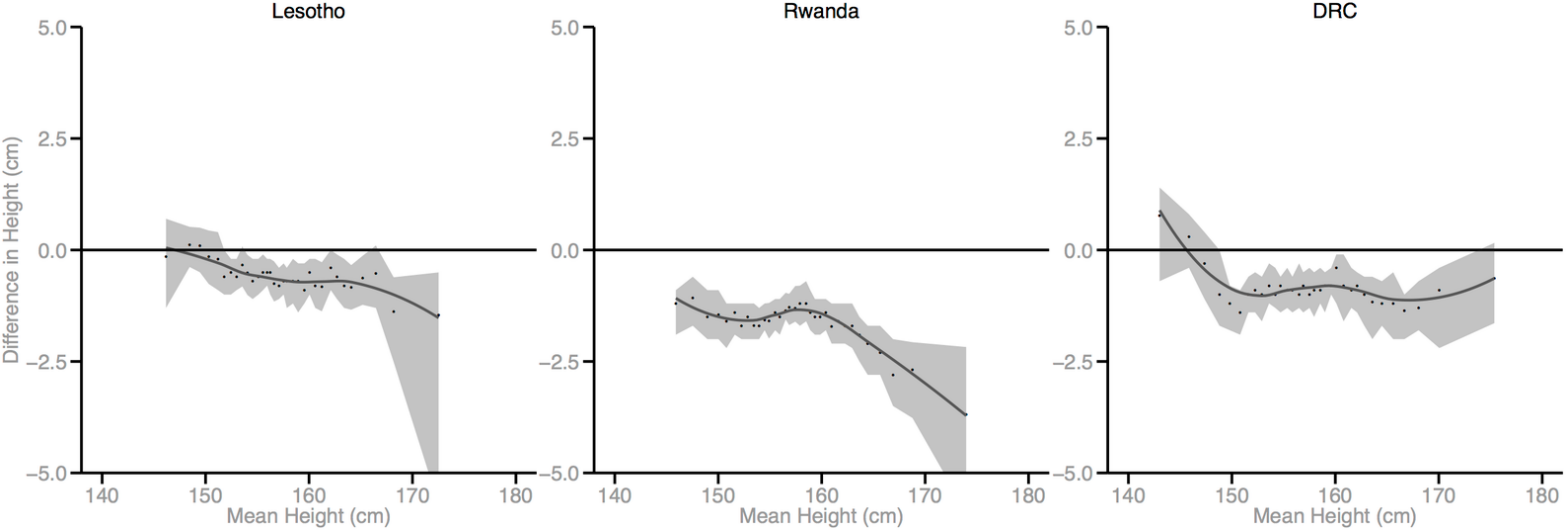
Mean-difference plots by country: Countries with decreased variance and decreased mean height (95% confidence interval shaded). DRC, Democratic Republic of the Congo.

**Fig 7:**
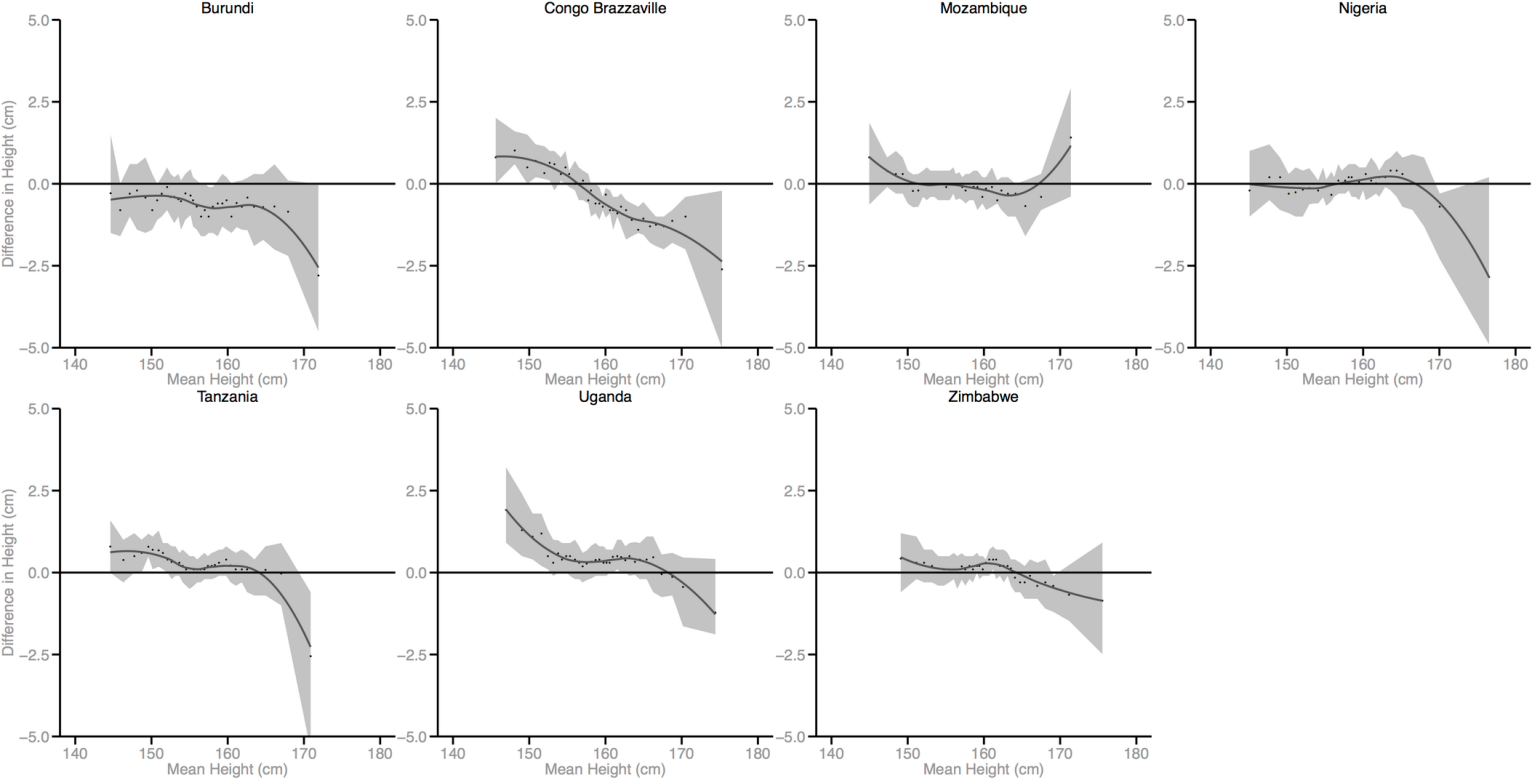
Mean-difference plots by country: Countries with decreased variance and no change in mean height (95% confidence interval shaded).

**Fig 8:**
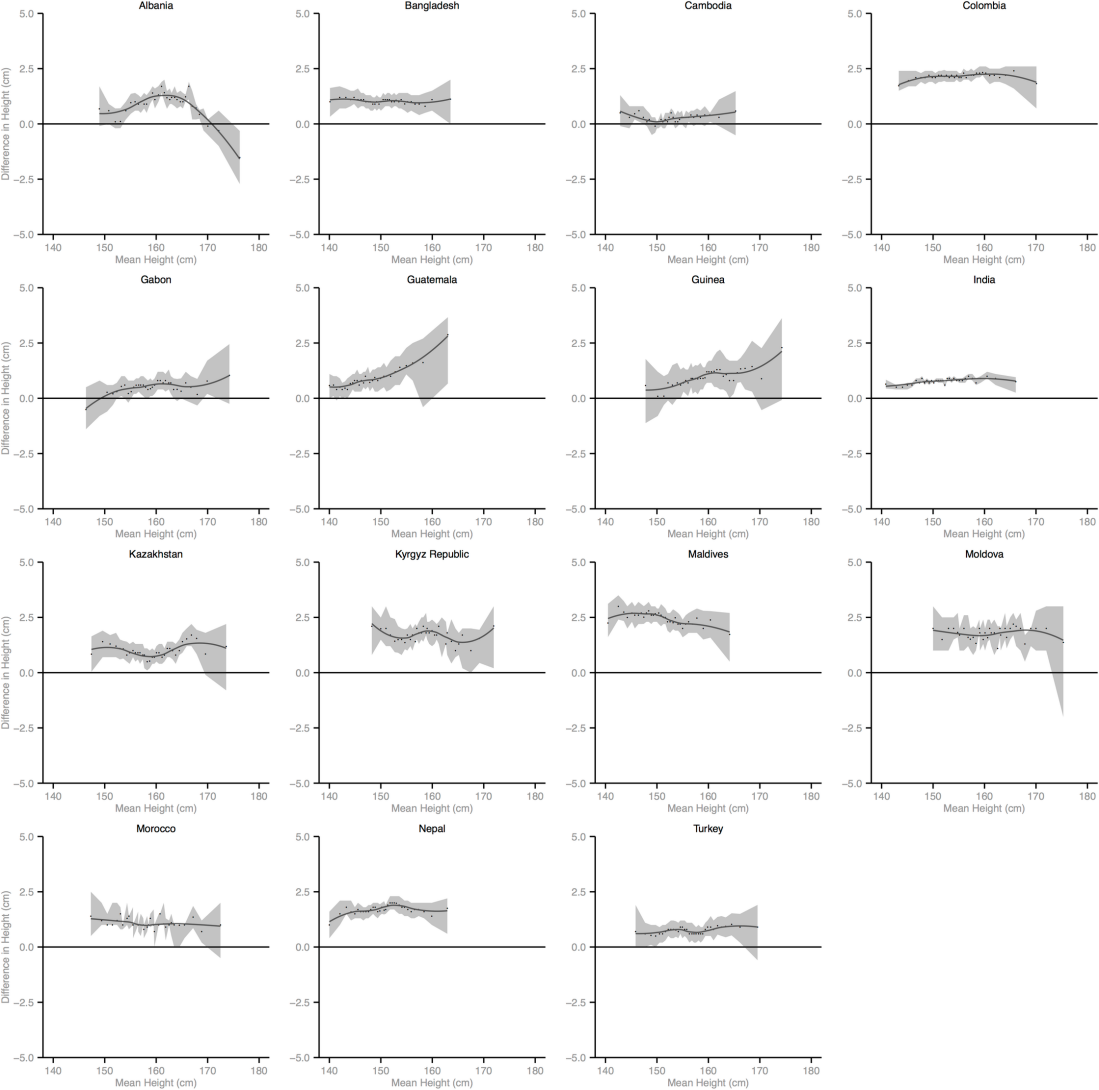
Mean-difference plots by country: Countries with no change in variance and increased mean height (95% confidence interval shaded).

**Fig 9:**
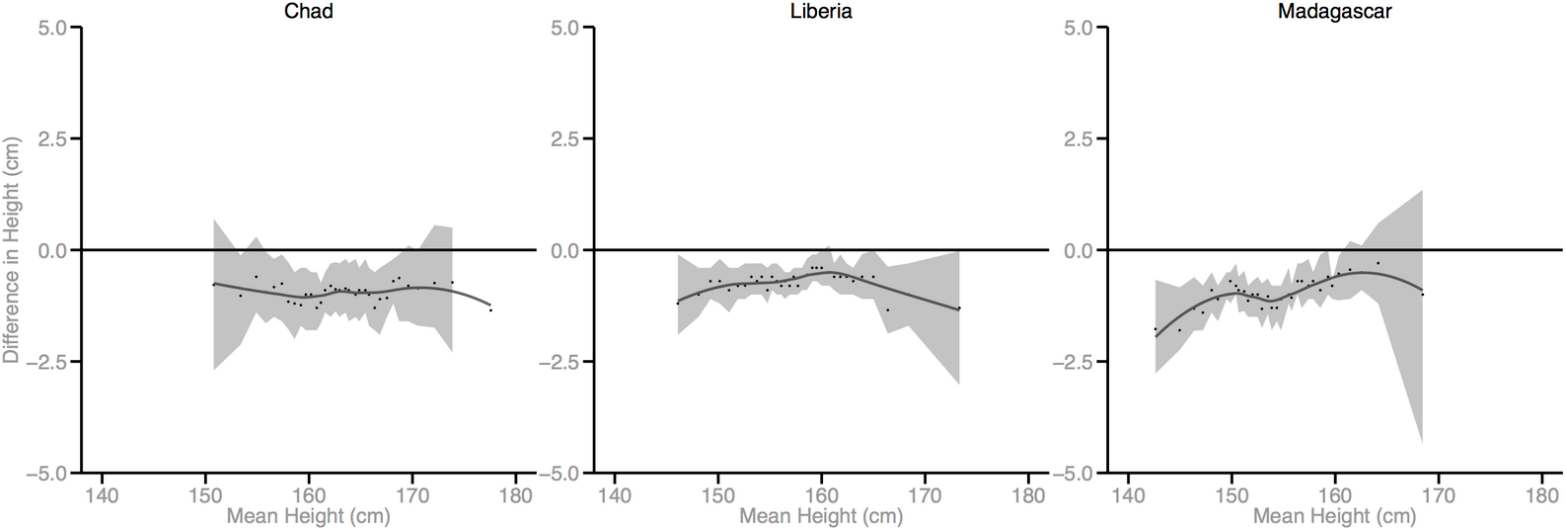
Mean-difference plots by country: Countries with no change in variance and decreased mean height (95% confidence interval shaded).

**Fig 10:**
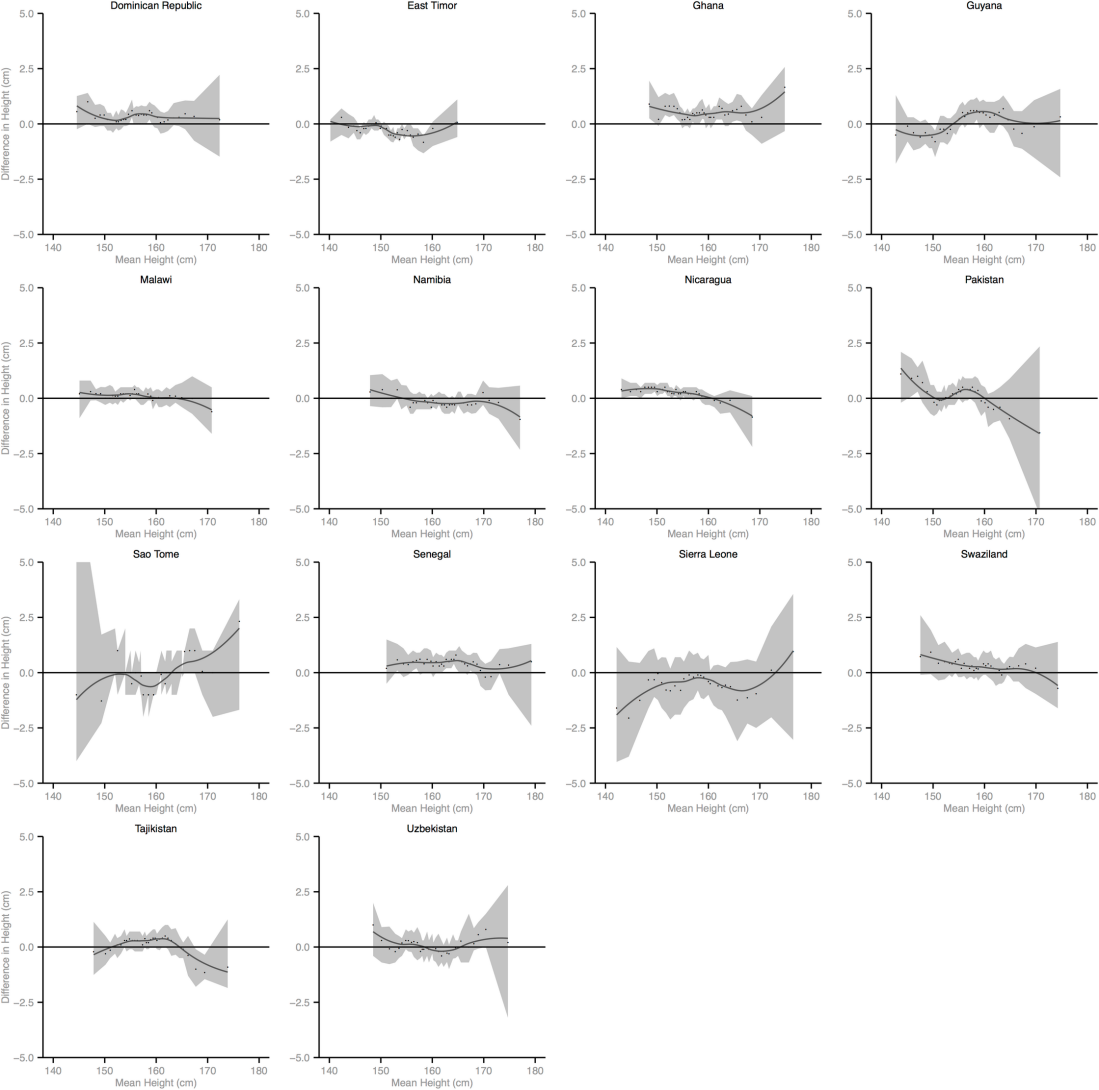
Mean-difference plots by country: Countries with no change in variance and no change in mean height (95% confidence interval shaded).

Figs 2–10 provide MD plots for all countries that experienced a significant change in the SD of height between the first and last birth cohort. Countries are grouped according to the direction of the change observed in terms of both mean height and the SD of height. Figs 2–4 include countries where variance was found to have increased, Figs 5–7 include countries where variance was found to have decreased, and Figs 8–10 include countries where we observed no change in the distribution between the first and last cohorts.

In countries with an increase in both the mean and the SD of height across birth cohorts, the gains in height appear to be primarily concentrated among the tallest segments of the distribution, with lesser gains observed among the shortest population segments (e.g., Benin). In countries where the mean increased but the SD decreased, the gains in height were greatest among the shortest segments of the population (e.g., Armenia).

Mali, Lesotho, Rwanda, and Democratic Republic of the Congo (DRC) all experienced decreases in mean height between the first and last birth cohorts; however, the distributional changes vary considerably. For Mali, the variance in height increased across birth cohorts. While all segments of the population lost height, the biggest losses occurred among the shortest segments. Conversely, for Lesotho, Rwanda, and DRC, the distribution contracted because the tallest population segments lost height. In Lesotho, despite the decreased mean, the shortest percentiles of the population gained height, in contrast to the large losses observed among the tallest percentiles.

Finally, among the 26 countries that exhibited no significant change in mean height, the distribution of height expanded in 4 countries and contracted in 7 countries. In the 4 countries where the distribution expanded, the shortest population segments lost height between the first and last birth cohorts while the tallest gained height (e.g., Cameroon). In the 7 countries that experienced a contraction in the distribution, gains were generally observed among the shortest population segments while the tallest either experienced losses in height or little change.

Multilevel regression models reveal that across all 59 countries, mean height did not have a significant relationship with SD (β = 0.015 cm, 95% CI: −0.032 cm, 0.061 cm). There was a significant relationship between median height and the tails of the distribution: for each 1-cm increase in median height, the 5th percentile of height increased by 0.915 cm (95% CI: 0.820 cm, 1.002 cm), and the 95th percentile of height by 0.995 cm (95% CI: 0.925 cm, 1.066 cm).

## Discussion

Our study has 3 salient findings. First, consistent with other studies, we find that less than half of the 59 LMICs included in our study have experienced gains in mean women’s height, while the majority have experienced no significant change, or even a decline, in mean height [34–36]. Second, we demonstrate that in many countries, the distribution of women’s height has not stayed constant across successive birth cohorts, and that variance does not have a consistent relationship with mean height changes. Multilevel models show that there is no evidence for an association between changes in mean height and SD in LMICs. Third, we find that a focus on the mean obscures distributional changes within countries.

The lack of significant change or declines in mean women’s height that we observed across countries may reflect inequitable progress globally in tackling some of the fundamental drivers of health disparities. Of the 26 countries that experienced increases in mean height, only 5 of them are in SSA while all 7 of the countries that experienced declines in mean height are in SSA. The pattern that we observed in attained height across birth cohorts among women is suggestive of either a stagnation or even deterioration in the environmental and nutritional circumstances that children in these countries faced through the 1990s [35] despite the fact that new medical technologies, political will, and stronger health systems have brought about substantial declines in infant mortality and acute childhood disease throughout SSA over the last several decades [64,65]. For example, the increased dispersion in women’s height observed in Cameroon may be a relic of the economic crisis and structural adjustment programs that the country experienced during the 1990s [66].

Our results also highlight the lack of a consistent relationship between changes in mean women’s height and its SD, which challenges the assumption that the mean is a useful summary measure of a population’s change in risk factors [48,49]. Reporting mean population change over time is a common way to track key public health indicators [36,42–45], and the mean is frequently used to measure success in reaching health and development goals [67,68]. The findings of this study suggest that caution should be used when reporting only the mean as a summary measure, especially when improving health equity is a goal. Several studies have interpreted increases in mean as an indication of an improvement in health and development conditions [38,69,70]; however, this interpretation is only valid at a population level following the assumption that the variance stays constant around the mean. In our study, 26 countries exhibited an overall increase in mean height across birth cohorts, but our examination of the variance provides a much more nuanced picture.

**Fig 11:**
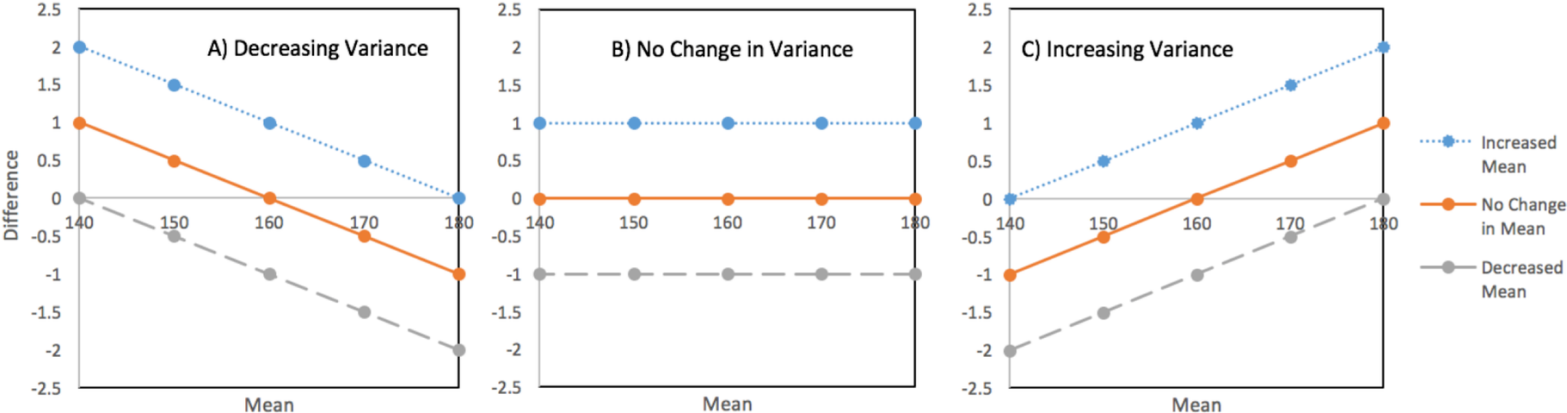
Theoretical models of patterns of change in mean and variance. (A) Decreased variance; (B) no change in variance; (C) increased variance.

In fact, the findings presented in this paper suggest that changes in the distribution of women’s height have occurred in all possible combinations of change in mean and variance (see Fig 1). The broad pattern observed suggests that in the countries where the distribution contracts, the shortest segments of the population generally do better than the tallest, whether that means more prominent gains or smaller losses in height. Conversely, where the variance increases, the tallest segments of the population generally fair better than the shortest. Fig 11 provides a smoothed representation of a graphical typology of the country-level changes that were observed.

Our results build on a recent study of 54 LMICs that suggests that height declined globally amongst the poorest wealth quintiles and increased amongst the richest wealth quintiles [35]. From a health equity standpoint, the increased dispersion that we see with regard to height may be suggestive of growing disparities in the distribution of environmental and nutritional inputs, especially in SSA. In Mali, the situation appears particularly bleak in that height has decreased across all population segments, but most markedly among the shortest segments.

Furthermore, our study points toward the possibility of growing disparities in exposures leading to adverse health outcomes, especially early-life episodes of noncommunicable diseases associated with loss of adult height. There remains a considerable research agenda in understanding the burden of these diseases among the most disadvantaged populations in LMICs, and little attention has been paid to identifying effective means of prevention and control among these population subgroups [71]. While there are strong arguments for population-based approaches that are meant to shift the entire distribution of risk factors downwards [2,72,73]—such as those that address inequities in access to economic opportunity, health care, education, and technology—the rising variance in some populations argues for the need for both population-wide and targeted interventions, such as nutrition programs targeting the children most at risk, to ensure both a rise in mean levels and a concomitant improvement in health equity [49].

Our study builds on a body of literature that examines height in relation to socioeconomic inequality [74]. Globally, a positive association has been found between mean height and a country’s Gini coefficient [74]. Additionally, the gap in potential versus attained height may be one way in which socioeconomic disparities are reproduced across generations. While several studies have found that adult height is likely to be at least partially determined by the economic conditions experienced during childhood [11,75–77], an individual’s height may in turn influence later socioeconomic potential [78–81] through school achievement, economic participation, and income generation [9,82–84]. Strauss and Thomas use historical data from Vietnam to argue that changes in the distribution of height provide insight into the changes in the distribution of resources [84]. They argue that prior to 1955, improvements in the distribution of resources in Vietnam led to considerable gains in mean height over time, which were characterized by the shortest segments of the population gaining more height than the tallest segments. However, after 1955, the improvements in mean height plateaued, and the distribution of height in the north began to expand—a phenomenon that they argue may reflect the disproportionate effects of war on the poor in the north.

Rising socioeconomic disparities, however, may not fully explain distributional changes for biological measures such as height. A recent analysis of changes in the BMI distribution in the US demonstrated that dispersion was increasing overall and within socioeconomic groups and suggested that other factors, such as assortative mating, social norms, and genetic predisposition, may play a role [49]. It is possible that some of these factors may also influence changes in the distribution of height. Another potential explanation for the widening dispersion that we observed relates to the possibility of there being some selection bias as a result of the historically adverse conditions found in many LMICs, which may have eliminated shorter individuals from earlier birth cohorts, leaving only the taller individuals, who were more likely to survive [12]. Moreover, recent advances in medical technology, including many of the same interventions that have led to the dramatic reductions in child mortality that have been observed across SSA in recent decades, raise the possibility that the phenomenon observed in our study is the result of increased survival among the shortest population segments in more recent birth cohorts. If shorter people are more likely to survive in more recent birth cohorts than in older birth cohorts, this would be a desirable scenario that would cause the distribution of height to widen. Finally, changes in ethnic diversity resulting from increased migration, such as refugee flows in some countries, may account for some of the changes observed in the relationship between the height parameters examined in this study.

The findings of this study should be interpreted in light of several limitations. First, women tend to lose height in old age; however, given the age range of the women in the study, we do not expect this to have played a significant role as height loss is expected to be minimal during the age range included in this study [35,85]. Second, DHS birth cohorts are not necessarily meant to be representative across birth cohorts, and thus averages may not be consistent over time across birth cohorts. To ensure that this did not influence our results, we examined each cohort to ensure that its mean remained similar as it advanced through survey waves. We found no notable differences in attained height comparing cohorts across survey years in this manner; thus, we do not believe that this would have had an effect on the findings. Third, while DHS uses extensive quality control procedures to ensure consistency in height measurements across surveys and years, there may be some small variation due to measurement error, but we do not expect this to have substantially influenced the results. Finally, the data used in this study include only women; thus, the results may not be fully generalizable to entire populations.

### Conclusions

Examining the height trends of populations using both the mean and the variance may enrich research on health equity [86], as adult height may serve as a link between early-life experiences and health in later life, including noncommunicable disease and longevity [36]. While increases in mean height may be indicative of progress in improving standards of living, such improvements have not occurred equitably in many countries. In several countries, despite marked improvements in mean women’s height overall, height among the shortest segments of the study population did not significantly change or even declined. In conclusion, the findings of this study indicate that the population distribution of women’s height does not necessarily stay constant in relation to the mean, and the overreliance on the mean as a summary measure may underestimate inequalities.

## Supporting information

S1 STROBE Checklist STROBE checklist. (DOC)

S1 Analysis Plan Analysis plan. (DOCX)

S1 Fig Residual plots (predicted versus residual estimates) and quantile–quantile (QQ) plots for the multilevel regression models for 59 DHS countries. (DOCX)

## Abbreviations

DHS: Demographic and Health Surveys
LMICs: low- and middle-income countries
MD: mean-difference
SSA: sub-Saharan Africa
