# Correction: Distributional change of women’s adult height in low- and middle-income countries over the past half century: An observational study using cross-sectional survey data

**DOI:** 10.1371/journal.pmed.1002588

**Published:** 2018-06-04

**Authors:** Jewel Gausman, Iván Mejía-Guevara, S. V. Subramanian, Fahad Razak

The second author's name is spelled incorrectly. The correct name is: Iván Mejía-Guevara. The correct citation is: Gausman J, Mejía-Guevara I, Subramanian SV, Razak F (2018) Distributional change of women’s adult height in low- and middle-income countries over the past half century: An observational study using cross-sectional survey data. PLoS Med 15(5): e1002568. https://doi.org/10.1371/journal.pmed.1002568

## References

[1] Gausman J, Meija Guevara I, Subramanian SV, Razak F (2018) Distributional change of women’s adult height in low- and middle-income countries over the past half century: An observational study using cross-sectional survey data. PLoS Med 15(5): e1002568 https://doi.org/10.1371/journal.pmed.1002568 29750787 10.1371/journal.pmed.1002568 PMC5947892

