# Supplementary material for: Distributional change of women’s adult height in low- and middle-income countries over the past half century: An observational study using cross-sectional survey data
Source: PLoS Med. 2018 May 11;15(5):e1002568. doi: 10.1371/journal.pmed.1002568 (PMC5947892; doi:10.1371/journal.pmed.1002568)
Supplement: S1 Analysis Plan — (DOCX) [file pmed.1002568.s002.docx]

**Background**

An individual’s maximum height attained during adulthood is both heritable and one that is heavily influenced by childhood environmental exposures (Silventoinen 2003) . Adverse circumstances during periods of rapid growth in childhood, such as low socioeconomic status, childhood illness, and nutritional deprivation, have been shown to be associated with decreased adult height attainment (Tucker-Seeley and Subramanian 2011),(Bozzoli, Deaton et al. 2009),(Steckel 1995). Adult height is also associated with future health and well-being. Studies have found that height is negatively associated with mortality from a variety of non-communicable diseases (Emerging Risk Factors Collaboration 2012) and declines in cognitive function (Case and Paxson 2008), and positively associated with future economic potential and earnings (Case and Paxson 2006).

Two recent studies have found that among low and middle income countries, countries in Africa have experienced significant declines in height across recent birth cohorts, while heights have generally increased in countries located in Europe, Eastern Mediterranean, South-East Asia and the Western Pacific (Akachi and Canning 2010),(Subramanian, Özaltin et al. 2011). Within countries, gains and losses in height are concentrated among socioeconomic groups. In a study examining height across birth cohorts in 54 low and middle income countries, declines in mean height across birth cohorts were found to be greater among the poorest wealth quintiles while gains in mean height were more pronounced among the wealthier quintiles (Subramanian, Özaltin et al. 2011). Such studies may point toward inequity in how changes in height attainment occur in populations over time.

The epidemiologist Geoffrey Rose argued that for most risk factors, such as blood pressure and cholesterol, as the average level in the population changed, the “dispersion around the average remains rather constant” (Rose, Khaw et al. 2008). While his findings suggested that this hypothesis also applied to BMI (Rose and Day 1990), studies have since found that weight gain tends to be concentrated among higher percentiles of the BMI distribution within a population (Razak, Corsi et al. 2013). Thus, a similar reliance on the mean as a summary measure of height may obscure significant changes in the distribution of height over time within and across populations that have important implications for health equity.

This study seeks to 1) describe changes in adult height across cohorts 2) assess whether changes in mean height are related to changes in the dispersion of height across birth cohorts within and across countries, 3) determine whether patterns exist with regard to which parts of the height distribution experience the greatest height gains or losses across birth cohorts.

**Methods**

**Data Sources**

Data for this analysis will obtained from the Demographic and Health Surveys (DHS), which are nationally representative, cross-sectional household surveys that have been conducted regularly in about 70 countries since 1984 (Rutstein 2006). DHS surveys are intended to be comparable over time and across countries. To achieve this, DHS employs standardized interviewer training, measurement tools, data collection techniques and questionnaires (ORC MACRO 2006). DHS ensures that they are nationally representative by employing a multistage stratified cluster design. Strata are defined by urban and urban locality, as well as by large administrative units, such as states or regions. During the first stage, the sampling frame is comprised by a complete list of primary sampling units (PSUs), developed based on unique geographic units (often census enumeration areas) that cover the entire country. PSUs are then selected within each stratum with probability proportional to size sampling (PPS) based on the number of units, meaning that each unit has a defined, equal probability of selection. Second, a listing of all households within a selected PSU is generated through field visits. From the list, a fixed sample of households is selected with equal systematic probability (ICF International 2012). The final sample size ranges from 5000 and 30 000 households, depending on the country (Corsi, Neuman et al. 2012).

**Study Population and Sample Size**

The study population will include women aged 25-49 available from survey waves spanning from 1993 to 2013. Women aged less than 25 will be excluded from the sample in order to ensure that full height attainment had been achieved. Women with missing height values or with implausible values recorded for height (less than 100cm or greater than 200 cm) will also be excluded from the study (Subramanian, Özaltin et al. 2011).

**Outcome and Exposure**

Attained height (measured in centimeters) will be specified as a continuous variable, and is considered to be accurate to 1 millimeter (Macro International Inc. 2008). The standard deviation (SD) and the 5^th^ and 95^th^ percentiles will also be used as outcomes in statistical modeling because they provide information on how the distribution of height changes between and within countries over time.

The primary exposure of interest was considered to be cohort. To compare women over time, women will be divided into birth cohorts ranging from 1950-59, 1960-69, 1970-79, and 1980-89 within each country.

**Analysis**

We will use a pooled dataset that includes all countries and survey waves. We will also create a second database also that includes summary percentile values and 33-quantile values for the height distribution in each country for each birth cohort. Stata v14.0 (StataCorp 2015) will be used to conduct the statistical analysis and graphics will be produced using the statistical package R (R Core Team 2013).

Research Question 1 will be addressed as follows. Summary tables that describe basic statistics of the population will be constructed according to country and birth cohort. These tables will include change in mean height and standard deviation of height for each country. An F-test for equality of variance will be performed to test the difference in SD between the earliest and most recent birth cohorts for each country.

Research Questions 2 and 3 will be addressed using both graphical and statistical approaches.

Graphical Analyses

Graphical approaches will be used to examine height within countries across birth cohorts. The first type of plot created will be the Quantile-Quantile (QQ) plot, which has previously been used to examine distributional changes in BMI (Razak, Corsi et al. 2013). QQ plots are particularly effective at depicting change at the tails of the distribution (Wilk and Gnanadesikan 1968). To construct the QQ plot, 33-quantiles (1^st^, 4^th^…94^th^, 97^th^) of height (y-axis) from the most recent birth cohort were plotted against the corresponding 33-quantiles of height (x-axis) from the earliest birth cohort for each individual country. 33- quantiles were used instead of percentiles in order to improve the smoothness of the graphical distributions. If the distribution of height remains the same across birth cohorts, regardless of overall gains or decreases in height within the country, the QQ plot will fall on the line of equality (line y=x). If segments of the QQ plot fall above the line, it indicates that certain segments of the population experienced disproportionate gains in height, while if the plot falls below the line of equality, it indicates that segments of the population experienced declines in height across birth cohorts.

The second plot type that will be used is the mean-difference plot. Mean difference plots graph the differences between corresponding quantiles on the y-axis against the means of the same quantile on the x-axis. The point’s location on the y-axis shows the direction and magnitude of the shift in relation to its difference from the horizontal line representing zero (Flegal and Troiano 2000). Mean-difference plot will be created for each country showing the shift in distribution of height between the most recent birth cohort and the earliest birth cohort available for each country. Points that fall below the line at zero indicate losses in height at that place in the distribution, while points that fall above the line indicate gains in height.

**Statistical Analyses**

Using data from all countries and cohorts, multilevel regression models will constructed to examine the association between 1) mean height and SD of height (research question 2) and 2) median height and the 5^th^ and 95^th^ percentiles of height (research question 3)

Multilevel regression will be used to account for the nested structure of the data, as the outcome measures of interest (SD, 5^th^ percentile or 95^th^ percentile) is nested within cohort *i* at level 1 which is nested in country *j* at level 2.

The general statistical model at level 1 for standard deviation of height will be specified as:

$$\boldsymbol{Y}_{\boldsymbol{ij}}\boldsymbol{=}\boldsymbol{B}_{\boldsymbol{0}\boldsymbol{j}}\boldsymbol{+}\boldsymbol{B}_{\boldsymbol{1}}\boldsymbol{heigh}\boldsymbol{t}_{\boldsymbol{ij}}\boldsymbol{+}\boldsymbol{e}_{\boldsymbol{ij}}$$

where *y*_ij_ is the standard deviation (SD) of height in cohort *i* in country *j*, β_0j_ is the overall SD of height in each country, β_1_ is the slope of the relationship between mean height and SD of height, *height*_ij_ is the mean height in cohort *i* in country *j,* and e_ij_ is the residual for each cohort within countries.

The model at level 2 is represented as:

$$\boldsymbol{B}_{\boldsymbol{oj}}\boldsymbol{=}\boldsymbol{B}_{\boldsymbol{0}}\boldsymbol{+}\boldsymbol{u}_{\boldsymbol{0}\boldsymbol{j}}$$

where β_0_ is the grand mean SD of height across all countries and *u*_0j_ is the residual of the mean SD of height for country *j* from the overall of SD of height. Both models can be combined to obtain:

$$\boldsymbol{Y}_{\boldsymbol{ij}}\boldsymbol{=}\boldsymbol{B}_{\boldsymbol{0}}\boldsymbol{+}\boldsymbol{B}_{\boldsymbol{1}}\boldsymbol{heigh}\boldsymbol{t}_{\boldsymbol{ij}}\boldsymbol{+(}\boldsymbol{u}_{\boldsymbol{0}\boldsymbol{j}}\boldsymbol{+}\boldsymbol{e}_{\boldsymbol{ij}}\boldsymbol{)}$$

**References**

Akachi, Y. and D. Canning (2010). "Health trends in Sub-Saharan Africa: conflicting evidence from infant mortality rates and adult heights." Economics & Human Biology **8**(2): 273-288.

Bozzoli, C., et al. (2009). "Adult height and childhood disease." Demography **46**(4): 647-669.

Case, A. and C. Paxson (2006). Stature and status: Height, ability, and labor market outcomes, National Bureau of Economic Research.

Case, A. and C. Paxson (2008). "Height, health, and cognitive function at older ages." The American Economic Review **98**(2): 463.

Corsi, D. J., et al. (2012). "Demographic and health surveys: a profile." International Journal of Epidemiology **41**(6): 1602-1613.

Emerging Risk Factors Collaboration (2012). "Adult height and the risk of cause-specific death and vascular morbidity in 1 million people: individual participant meta-analysis." International Journal of Epidemiology.

Flegal, K. M. and R. P. Troiano (2000). "Changes in the distribution of body mass index of adults and children in the US population." International journal of obesity **24**(7): 807-818.

ICF International (2012). Demographic and Health Survey Sampling and Household Listing Manual. Calverton, Maryland, Measure DHS.

Macro International Inc. (2008). Anthropometry, anemia, and HIV testing field manual. Calverton, Maryland.

ORC MACRO (2006). Demographic and health survey interviewer’s manual. Calverton, Maryland, ORC Macro.

R Core Team (2013). R: A language and environment for statistical computing. Vienna, Austria, Foundation for Statistical Computing.

Razak, F., et al. (2013). "Change in the body mass index distribution for women: analysis of surveys from 37 low-and middle-income countries." PLoS Med **10**(1): e1001367.

Rose, G. and S. Day (1990). "The population mean predicts the number of deviant individuals." Bmj **301**(6759): 1031-1034.

Rose, G. A., et al. (2008). Rose's strategy of preventive medicine: the complete original text, Oxford University Press, USA.

Rutstein, S. O. (2006). "Guide to DHS statistics."

Silventoinen, K. (2003). "Determinants of Variation in Adult Body Height." Journal of Biosocial Science **35**(02): 263-285.

StataCorp (2015). Stata Statistical Software: Release 14. College Station, TX, StataCorp LP.

Steckel, R. H. (1995). "Stature and the Standard of Living." Journal of economic literature **33**(4): 1903-1940.

Subramanian, S., et al. (2011). "Height of nations: a socioeconomic analysis of cohort differences and patterns among women in 54 low-to middle-income countries." PloS one **6**(4): e18962.

Tucker-Seeley, R. D. and S. Subramanian (2011). "Childhood circumstances and height among older adults in the United States." Economics & Human Biology **9**(2): 194-202.

Wilk, M. B. and R. Gnanadesikan (1968). "Probability plotting methods for the analysis for the analysis of data." Biometrika **55**(1): 1-17.
