# Supplementary material for: Distributional change of women’s adult height in low- and middle-income countries over the past half century: An observational study using cross-sectional survey data
Source: PLoS Med. 2018 May 11;15(5):e1002568. doi: 10.1371/journal.pmed.1002568 (PMC5947892; doi:10.1371/journal.pmed.1002568)
Supplement: S1 Fig — (DOCX) [file pmed.1002568.s003.docx]

S1 Fig. Residual plots (predicted VS. residual estimates) and quantile-quantile (QQ) plots

for the Multilevel regression models for 59 DHS countries
